# Molecular landscape and efficacy of HER2-targeted therapy in patients with HER2-mutated metastatic breast cancer

**DOI:** 10.1038/s41523-020-00201-9

**Published:** 2020-10-30

**Authors:** Zongbi Yi, Guohua Rong, Yanfang Guan, Jin Li, Lianpeng Chang, Hui Li, Binliang Liu, Wenna Wang, Xiuwen Guan, Quchang Ouyang, Lixi Li, Jingtong Zhai, Chunxiao Li, Lifeng Li, Xuefeng Xia, Ling Yang, Haili Qian, Xin Yi, Binghe Xu, Fei Ma

**Affiliations:** 1 Department of Medical Oncology, National Cancer Center/National Clinical Research Center for Cancer/Cancer Hospital, Chinese Academy of Medical Sciences and Peking Union Medical College, Beijing, 100021 China; 2 Geneplus-Beijing Institute, Beijing, China; 3 Department of Computer Science and Technology, School of Electronic and Information Engineering, Xi’an Jiaotong University, 28 West Xianning Road, Xi’an, Shanxi 710048 China; 4 Department of Breast Cancer Medical Oncology, Hunan Cancer Hospital, Changsha, 410013 China; 5 State Key Laboratory of Molecular Oncology, National Cancer Center/National Clinical Research Center for Cancer/Cancer Hospital, Chinese Academy of Medical Sciences and Peking Union Medical College, Beijing, 100021 China

**Keywords:** Breast cancer, Targeted therapies

## Abstract

Human epidermal growth factor receptor 2 (HER2) protein overexpression or gene amplification is an important predictive biomarker for identifying patients with breast cancer, who may benefit from HER2-targeted therapy. However, little is known about the molecular landscape and efficacy of HER2-targeted therapy in patients with HER2-mutated metastatic breast cancer. We analysed the HER2 mutation features of 1184 patients with invasive breast cancer. In addition, a single-arm, prospective, phase-II study (NCT03412383) of pyrotinib was conducted in patient with metastatic HER2 amplification-negative, mutation-positive breast cancer. Peripheral blood was collected from each patient and circulating tumour DNA (ctDNA) sequencing was performed using a 1021 gene panel. HER2 mutations were detected in 8.9% (105/1184) of patients. The HER2 amplification-positive patients had a higher mutation frequency than the HER2 amplification-negative patients (19.5% vs. 4.8%, *P* < 0.001). A multivariate Cox regression analysis indicated that patients with HER2 mutations had a shorter progression-free survival (PFS) than HER2 wild-type patients (median PFS 4.7 months vs. 11.0 months, hazard ratio 2.65, 95% confidence interval 1.25–5.65, *P* = 0.011). Ten HER2 amplification-negative, mutation-positive patients who received pyrotinib monotherapy were ultimately included in the efficacy analysis. The median PFS was 4.9 months. The objective response rate (complete response + partial response) was 40.0% and the clinical benefit rate (complete response + partial response + stable disease over 24 weeks) was 60%. In conclusion, a HER2 gene mutation analysis is potentially useful to identify biomarkers of trastuzumab resistance in HER2 amplification-positive patients. Patients with HER2-mutated, non-amplified metastatic breast cancers may benefit from pyrotinib.

## Introduction

Human epidermal growth factor receptor 2 (HER2, also known as *ERBB2*) amplification or overexpression is detected in 20–30% of patients with breast cancer and is associated with a poor prognosis^1,2^. Overexpression of the HER2 protein or amplification of the HER2 gene is an important predictive biomarker for identifying patients with breast cancer, who may benefit from HER2-targeted therapy. The mechanisms of HER2 activation include not only the overexpression of the HER2 protein and amplification of the HER2 but also somatic mutations in HER2, leading to activation of the HER2 gene^3,4^. Next-generation sequencing has indicated that somatic mutations in HER2 are present in ~2–5% of primary breast cancers^3,5–9^. Most HER2 somatic mutations have been reported in HER2 amplification-negative breast cancers^10^. However, previous studies of HER2 mutations have mainly focused on early-stage breast cancer based on primary tissues. Little is known about the molecular landscape and efficacy of HER2-targeted therapy in patients with HER2-mutated metastatic breast cancer.

HER2 amplification-positive patients might benefit from existing HER2-targeted drugs, but some HER2 mutations result in resistance to the same treatments^6,11–13^. However, HER2 amplification-negative patients were reported to have benefited from anti-HER2 therapies when they carried activating somatic mutations in HER2^4,8,14^. The effects of HER2 mutations on HER2 amplification-positive and HER2 amplification-negative breast cancers may differ. The effect of HER2 mutations on the likelihood of a response to HER2-targeted therapies in patients with different molecular subtypes of breast cancer is particularly to understand, which might help us choose more precise HER2-targeted therapies.

Therefore, we used targeted capture sequencing to analyse circulating tumour DNA (ctDNA) to determine the frequency and spectrum of HER2 mutations in patients with advanced breast cancer and to analyse the relationships between HER2 mutations and their effects on the efficacy of anti-HER2 therapies.

## Results

### Patient characteristics and HER2 mutation prevalence

In total, 1184 metastatic breast cancer patients were enrolled in the retrospective cohort. HER2 mutations were detected in 8.9% (105/1184) of patients. The HER2 amplification-positive patients had a higher mutation frequency than the HER2-negative patients (19.5%, 64/329 vs. 4.8%, 41/855; *P* < 0.001). The differences in the major clinical characteristics of the patients with HER2 mutations and patients without HER2 mutations are outlined in Table 1.

**Table 1:** Patient characteristics.

Characteristics	No. of patients (%)			*P*
	Total (*n* = 1184)	HER2 wild-type (*n* = 1079)	HER2 mutant (*n* = 105)	
Age at initial diagnosis				
≤35 Years	171 (14.4)	151 (14.0)	20 (19.0)	0.160
>35 Years	1013 (85.6)	928 (86.0)	85 (81.0)	
Histopathological				
Invasive ductal carcinoma	1049 (88.6)	962 (89.2)	87 (82.9)	0.169
Invasive lobular carcinoma	45 (3.8)	38 (3.5)	7 (6.7)	
Mixed invasive ductal and invasive lobular	2 (0.2)	2 (0.2)	0 (0.0)	
Other	88 (7.4)	77 (7.1)	11 (10.5)	
HR status				
Positive	696 (58.8)	635 (58.9)	61 (58.1)	0.182
Negative	422 (35.6)	380 (35.2)	42 (40.0)	
Unknown	66 (5.6)	64 (5.9)	2 (1.9)	
HER2 status				
Positive	329 (27.8)	265 (24.6)	64 (61.0)	<0.001
Negative	855 (72.2)	814 (75.4)	41 (39.0)	
Molecular subtype				
HR+/HER2−	522 (44.1)	488 (45.2)	34 (32.4)	<0.001
HR+/HER2+	174 (14.7)	147 (13.6)	27 (25.7)	
HR−/HER2+	155 (13.1)	118 (10.9)	37 (35.2)	
HR−/HER2−	267 (22.6)	262 (24.3)	5 (4.8)	
Unknown	66 (5.6)	64 (5.9)	2 (1.9)	

*HER2* human epidermal growth factor receptor 2, *HR* hormone receptor.

**Fig. 1:**
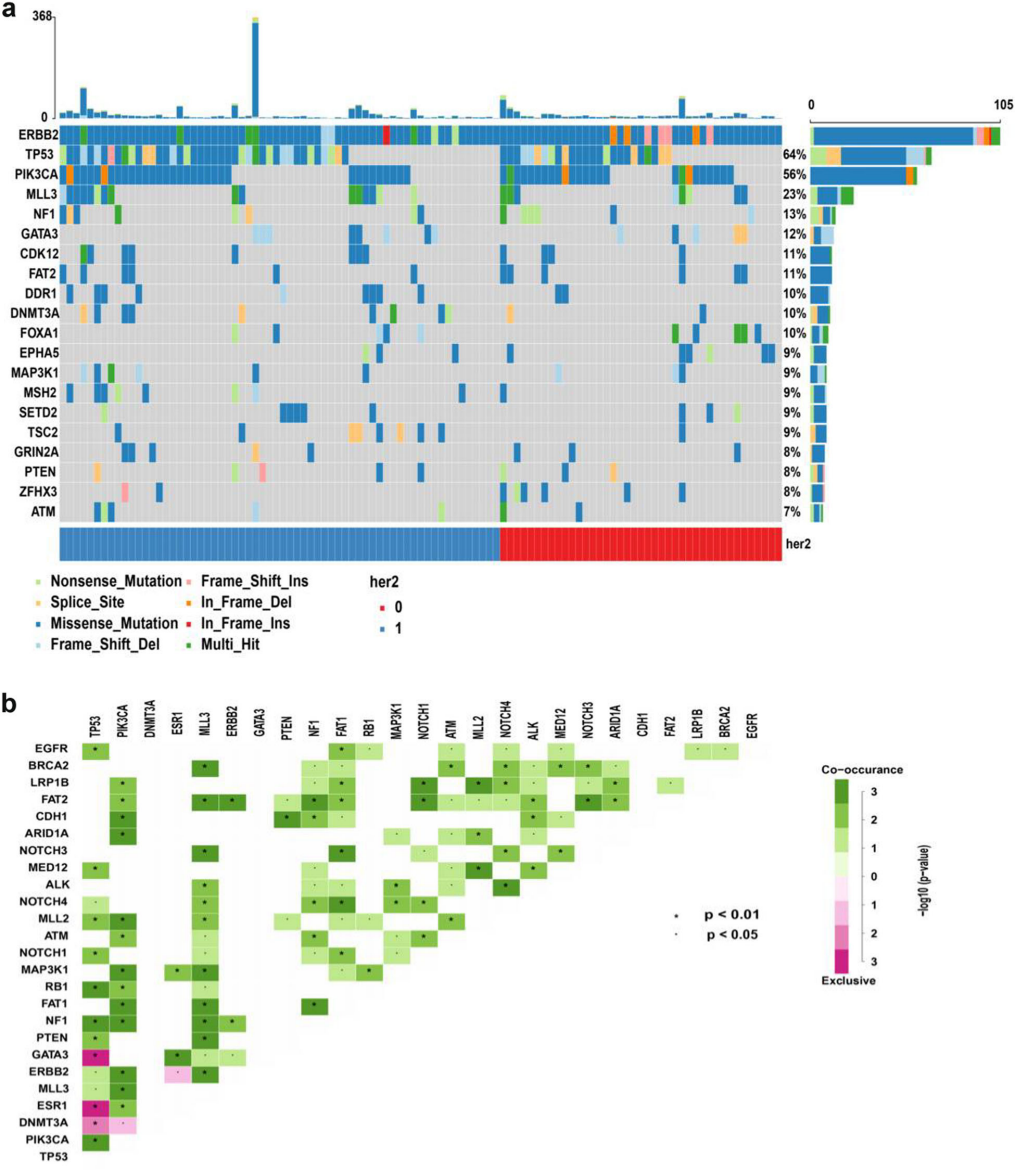
Somatic mutation spectrum and interaction of top 20 genes. **a** Mutation spectrum of the top 20 genes in 105 patients carrying HER2 mutations. HER2 = 0 defined as HER2 amplification negative and HER2 = 1 defined as HER2 amplification positive. **b** Somatic interactions of the top 20 genes. Pairwise Fisher’s exact test was used to detect significant gene pairs. HER2, human epidermal growth factor receptor 2.

We identified 529 HER2 somatic mutations, including 386 different mutant sites, in 105 patients (Fig. 1a and Supplementary Data 1). The average depth of coverage of the target genes was 1950-fold. A total of 29.3% (113/386) mutations have been recorded in the COSMIC database (https://cancer.sanger.ac.uk/cosmic) and/or cBioPortal database (http://www.cbioportal.org). Most of the mutations (92.2%, 488/529) were missense mutations, 3.6% (19/529) mutations were nonsense mutations and 2.8% (15/529) were frameshift mutations. HER2 p.V777L, p.L755S and p.D769Y were the top three mutations by frequency and were detected in 13 (12.4%), 10 (9.5%) and 10 (9.5%) patients, respectively. The extracellular portion of HER2 was most commonly mutated (42.5%, 225/529), followed by the kinase domain (28.9%, 153/529), tail (20.2%, 107/529) and transmembrane/juxtamembrane (4.3%, 23/529) domains. Importantly, 5.9% (31/529), 7.2% (38/529) and 5.1% (27/529) of mutations located in HER2 exons 19, 20 and 21, respectively. Notably, 95.5% (505/529) of HER2 mutations occurred in subclonal tumours. Only two patients (1.9%) had a single detected HER2 mutation; the other 103 (98.1%) patients had HER2 mutations along with mutations in other genes. The most frequent co-occurring mutations identified in the genes were *TP53*, *PIK3CA*, *MLL3*, *FAT2*, *NF1* and *GATA3* genes. However, *ESR1* and HER2 mutations were mutually exclusive (Fig. 1b). We used Cancer Genome Interpreter (https://eur02.safelinks.protection.outlook.com/?url=https%3A%2F%2Fwww.cancergenomeinterpreter.org%2Fhome&data=02%7C01%7Csineadtoomey@rcsi.ie%7Cfc7416f999a3484c78eb08d7928065c4%7C607041e7a8124670bd3030f9db210f06%7C0%7C0%7C637138952481122708&sdata=RyWLsWiAUGzxfS%2BLx%2FOtL5rvPuTP%2Bus4vGD%2B6KBlZ5A=&reserved=0) to identify total of 386 *ERBB2* unique variants in our study. The result showed 194 variants were predicted as driver mutations and 192 variants were predicted as passenger mutations. The top three frequency mutations of HER2 p.V777L, p.L755S and p.D769Y were all predicted as driver mutations.

### HER2 mutations in patients stratified according to the HER2 amplification status

**Fig. 2:**
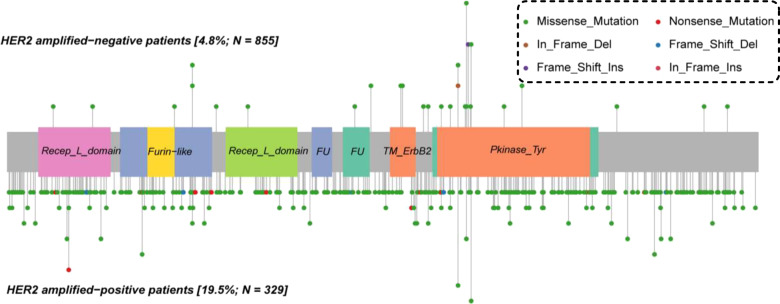
Distribution of HER2 variants in 105 patients with breast cancer stratified according to the HER2 amplification status. HER2, human epidermal growth factor receptor 2.

We further analysed HER2 mutations in patients stratified according to the HER2 amplification status. A total of 46.2% (24/52) of HER2 mutations were located in exon 19 or 20 in HER2 amplification-negative patients, a value that was significantly higher than the 9.4% (45/477) of HER2 mutations detected in HER2 amplification-positive patients (*P* < 0.001). HER2 mutations in HER2 amplification-positive patients more likely occur in the extracellular domain than in HER2 amplification-negative patients (44.2%, 212/447 vs. 25.0%, 13/52; *P* = 0.002), in whom the mutation were more likely to occur in the HER2 kinase domain (57.7%, 30/52 vs. 25.8%, 123/477; *P* < 0.001). A greater percentage of frameshift mutations in HER2 was detected in HER2 amplification-negative patients than that in HER2 amplification-positive patients (13.5%, 7/52 vs. 1.7%, 8/477; *P* < 0.001). Notably, 96.9% (462/477) of HER2 mutations occurred in subclonal tumours from HER2 amplification-positive patients, which was greater than the value of 82.7% (43/52) observed in HER2 amplification-negative patients (*P* < 0.001). HER2 mutations in patients with HER2 amplification-negative breast cancer had a higher frequency of HER2 hotspot mutations than in patients with HER2 amplification-positive breast cancer (36.5%, 19/52 vs. 5.0%, 24/477; *P* < 0.001). The HER2 mutation features in patients stratified according to the HER2 amplification status are shown in Table 2 and Fig. 2.

**Table 2:** Characteristics of HER2 mutations in patients stratified according to the HER2 amplification status.

Characteristics	No. of cases (%)			*P*
	Total (*n* = 529^a^)	HER2 amplification-positive (*n* = 477)	HER2 amplification-negative (*n* = 52)	
ERBB2 exon				
19	31 (5.9)	17 (3.6)	14 (26.9)	<0.001
20	38 (7.2)	28 (5.9)	10 (19.2)	
21	27 (5.1)	25 (5.2)	2 (3.8)	
Other	433 (81.9)	407 (85.3)	26 (50.0)	
Mutation site				
ECD	225 (42.5)	212 (44.4)	13 (25.0)	<0.001
TKI	153 (28.9)	123 (25.8)	30 (57.7)	
Other	151 (28.5)	142 (29.8)	9 (17.3)	
Variant classification				
Missense	488 (92.2)	443 (92.9)	45 (86.5)	<0.001
Nonsense	19 (3.6)	19 (4.0)	0 (0.0)	
Frame shift	15 (2.8)	8 (1.7)	7 (13.5)	
Other	7 (1.3)	7 (1.5)	0 (0.0)	
Variant type				
SNP	514 (97.2)	469 (98.3)	45 (86.5)	<0.001
INS	6 (1.1)	2 (0.4)	4 (7.7)	
DEL	9 (1.7)	6 (1.3)	3 (5.8)	
VAF mean ± SD	2.1 ± 9.0	1.2 ± 7.0	10.2 ± 17.4	<0.001
Clonality status				
Clonal	24 (4.5)	15 (3.1)	9 (17.3)	<0.001
Subclonal	505 (95.5)	462 (96.9)	43 (82.7)	
Hotspot mutations				
p.V777L	13 (2.5)	9 (1.9)	4 (7.7)	<0.001
p.D769Y/H	11 (2.1)	4 (0.8)	7 (13.5)	
p.L755S	10 (1.9)	7 (1.5)	3 (5.8)	
p.S310F/Y	9 (1.7)	4 (0.8)	5 (9.6)	
Other	486 (91.9)	453 (95.0)	33 (63.5)	

*DEL* deletion mutation, *ECD* extracellular domain, *HER2* human epidermal growth factor receptor 2, *INS* insertion mutation, *SNP* single-nucleotide polymorphism, *TKI* tyrosine kinase domain, *VAF* variant allele frequency.

^a^Statistical analysis based on the mutation number.

### Clinical outcomes of trastuzumab therapy in HER2 amplification-positive patients harbouring HER2 mutations

**Fig. 3:**
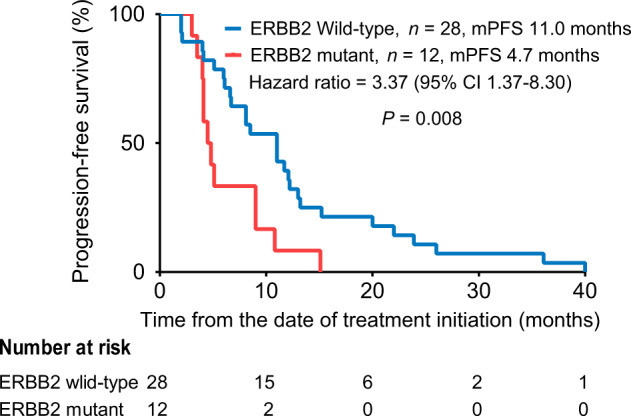
PFS HER2 amplification-positive patients who received trastuzumab as the first-line treatment and were stratified based on the HER2 mutation status. PFS progression-free survival, HER2 human epidermal growth factor receptor 2.

Forty HER2 amplification-positive patients who received trastuzumab combined with chemotherapy as the first-line therapy after being diagnosed with metastatic breast cancer were ultimately eligible for the analysis of progression-free survival (PFS). Twelve of the 40 patients carried HER2 somatic mutations. The median PFS of patients with HER2 mutations was 4.7 months compared with 11.0 months in patients with non-HER2 mutations [hazard ratio 3.37, 95% confidence interval (CI) 1.37–8.30, *P* = 0.008, Fig. 3]. According to the multivariate Cox regression analysis, patients with HER2 mutations had a shorter PFS than patients with a wild-type status (hazard ratio 2.65, 95% CI 1.25–5.65, *P* = 0.011; Supplementary Table 1). The PFS of 10 patients with HER2 mutations was <6 months.

### Clinical outcomes of pyrotinib therapy in HER2 amplification-negative patients harbouring HER2 mutations

**Fig. 4:**
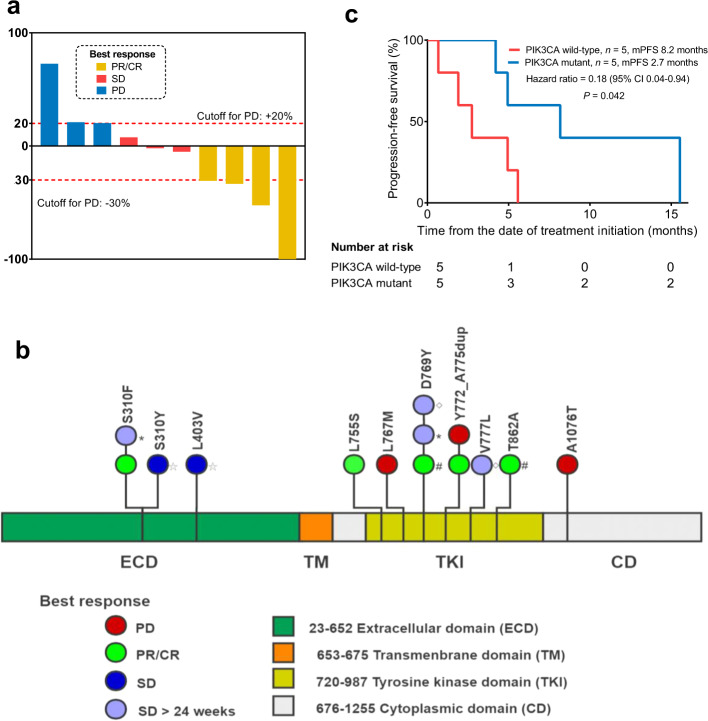
Clinical outcomes of pyrotinib therapy in HER2 amplification-negative patients harbouring HER2 mutations. **a** Maximum reduction in target lesions from baseline for HER2 amplification-negative, mutation-positive patients treated with pyrotinib. **b** Distribution of HER2 mutations in ten patients treated with pyrotinib. The symbols ⬦, *, # and ^☆^ represent concurrent HER2 mutations in the same patient. **c** PFS of pyrotinib-treated HER2 amplification-negative, mutation-positive patients stratified according to the *PIK3CA* mutation status.

Thirteen patients received pyrotinib monotherapy. Three withdrew from the study because of intolerance/adverse events (AEs) before the first evaluation of the tumour response. Therefore, ten patients were ultimately enrolled in the efficacy analysis. The median number of anti-cancer therapy regimens for metastatic breast cancer received prior to study entry was three (range, 2–10). The median PFS was 4.9 months (95% CI: 3.8 to 6.0 months) for all ten patients. One patient (10.0%) experienced a complete response (CR). Three patients (30.0%) experienced a partial response (PR) as the best response, three patients (30.0%) had a best response of stable disease (SD), and three patients (30.0%) had progressive disease (Fig. 4a). The objective response rate (ORR, CR + PR) was 40.0%. The clinical benefit rate (CBR, CR + PR + SD over 24 weeks) was 60%. The characteristics of these ten patients are described in Supplementary Table 2 and the grades of all major treatment-related AEs are presented in Supplementary Table 3.

**Fig. 5:**
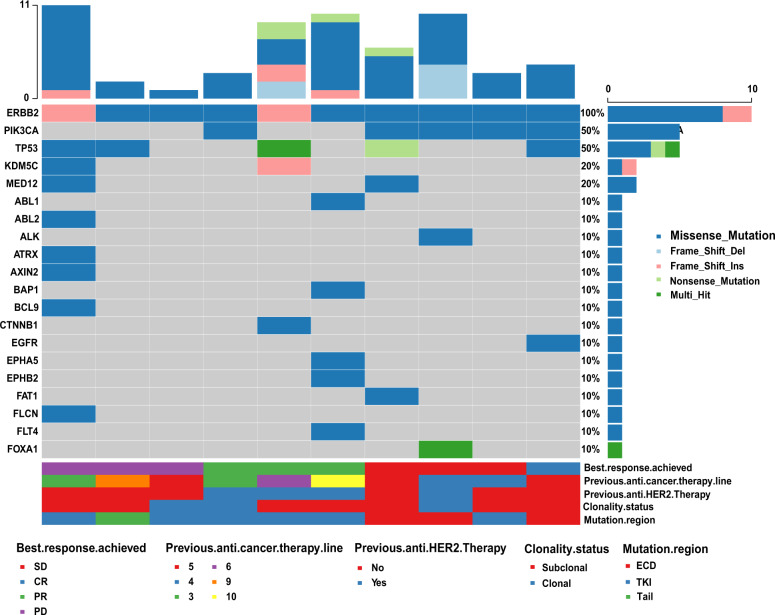
Mutational spectrum of the top 20 genes in 10 HER2 amplification-negative patients treated with pyrotinib. CR complete response, ECD extracellular domain, PD progressive disease, PR partial response, SD stable disease, TK tyrosine kinase domain.

We further analysed the association of genome feather and pyrotinib efficacy (Figs 4 and 5). Patients with *PIK3CA* mutations had a longer PFS than patients with wild-type *PIK3CA*. The median PFS of patients with *PIK3CA* mutations was 8.2 months compared with 2.7 months for patients with wild-type *PIK3CA* (hazard ratio 0.18, 95% CI 0.04–0.94, *P* = 0.042; Fig. 4c). We also analysed the co-occurrence of mutations in other HER family members. One patient (10%) who experienced a CR carried an epidermal growth factor receptor (EGFR) mutation. HER3 and HER4 mutations were not detected in all ten patients.

## Discussion

Next-generation sequencing-based assays indicate that HER2 somatic mutations are present in approximately 2–5% of primary breast cancers^10,15^. The vast majority of HER2 somatic mutations have been reported in HER2 amplification-negative patients^10^. Only a few mutations have been reported in patients with HER2 amplification-positive breast cancer. In the present study, the frequency of HER2 mutations in patients with metastatic breast cancer was 8.9%, which is higher than previously reported frequencies in other studies^7,10^. One of the explanations for this discrepancy is that all patients enrolled in the present study had metastatic breast cancer with a high tumour burden and had received multiple anti-cancer therapies. Most of the HER2 mutations (95.5%) occurred in the subclone and more likely co-occurred with mutations in other genes such as *TP53*, *PIK3CA*, *MLL3*, *FAT2*, *NF1* and *GATA3*. Therefore, we hypothesized that HER2 mutations might be induced by anti-cancer therapies. According to a recent study, HER2 mutations were more frequently observed in patients with advanced breast cancer compared with patients with early-stage tumours and HER2-activating mutations were induced by endocrine therapy^16^. Another previous study compared HER2 mutations in 18 pairs of primary and metastatic tumours, and found that drug-resistant HER2 mutations were enriched in metastatic tumours^7^. Both of these studies support our hypothesis that HER2 mutations might be acquired from anti-cancer therapies, but the mechanism requires further investigation. The current study used ctDNA to detect HER2 mutations and probably explains why we predominantly detected HER2 mutations in subclones. Interestingly, *ESR1* and HER2 mutations were mutually exclusive. The mechanism was unclear and requires further study. Another explanation for the high HER2 mutation frequency in the present study is that many previous studies detected only hotspot mutations in HER2. Nevertheless, this approach may miss multiple HER2 mutations, because the distribution of HER2 mutation sites is not concentrated. In addition, the current study used ctDNA to detect HER2 mutations in a large cohort of patients. Most previous studies detected HER2 mutations in tumour tissues. The use of ctDNA may overcome the tissue heterogeneity and has the potential to detect more mutations than a tissue biopsy from single organ. Two groups reported an enrichment of HER2 mutations in ctDNA from patients treated with endocrine therapy ^17,18^.

Furthermore, the HER2 mutation feature varied with the HER2 amplification status. The variant allele frequency of HER2 mutations in HER2 amplification-positive patients was significantly higher than in HER2 amplification-negative patients. The HER2 gene copy number gain might influence the variant allele frequency of HER2 mutations in HER2 amplification-positive patients. Moreover, HER2 mutations were more likely to occur in a subclone in HER2 amplification-positive patients than in HER2 amplification-negative patients. HER2 mutations were more likely to occur in the extracellular domain in HER2 amplification-positive patients and the mutations were more likely to occur in the HER2 kinase domain in HER2 amplification-negative patients. Based on these results, we surmised that HER2 mutations might also be induced by HER2-targeted therapy. HER2 mutations were recently shown to be induced by HER2-targeted therapy^19^. Patients with HER2 mutations and amplification were resistant to trastuzumab in the present study. Cocco et al.^20^ have reported in 2018 that six patients with tumours bearing coincident HER2 amplification and mutations, who received extensive pretreatments subsequently, exhibited a statistically significant response to neratinib monotherapy. However, randomized clinical studies are needed for further validation.

For HER2 amplification-negative patients, HER2 mutations were more likely to occur in the HER2 kinase domain and have a high variant allele frequency. Thus, HER2 mutations may be driver events in HER2 amplification-negative patients. A previous basic science study reported seven activating mutations—G309A, D769H, D769Y, V777L, P780ins, V842I and R896C—indicating sensitivity to the irreversible kinase inhibitor neratinib^4^. Chumsri et al.^21^ reported a HER2-negative patient who experienced a prolonged response to trastuzumab when it was administered in combination with pertuzumab and fulvestrant. Further molecular analyses revealed that the HER2 S310F mutation was present in the tumours. A phase-II clinical trial assessed the efficacy of neratinib in patients with HER2 mutation-positive amplification-negative metastatic breast cancer, and the CBR was 31% and the median PFS was 3.7 months^14^. We also conducted a clinical trial to explore the efficacy of pyrotinib in patients with HER2 amplification-negative, mutation-positive metastatic breast cancer. The median PFS was 4.9 months and the CBR was 60%. One patient with both HER2 and EGFR mutations achieved a CR to pyrotinib. Pyrotinib also targeted EFGR and thus patients with EGFR mutations may benefit from pyrotinib therapy. A previous study indicated that patients with EGFR mutations respond to neratinib^22^. In addition, the AE profile in this study was similar to that of previous studies^23,24^. Consistent with previous reports, diarrhoea was the most frequent treatment-related AE. Most of the AEs were tolerable.

*PIK3CA* mutations were reported to be a negative predictor of the response to HER2-targeted therapy in patients with HER2 amplification-positive breast cancer^25,26^. In the SUMMIT study^8^, the activation of the phosphatidyl inositol 3-kinase/AKT/mammalian target of rapamycin pathway did not adversely affect the likelihood of the clinical benefit of neratinib in patients with various cancers. In the present study, HER2-mutated, non-amplified patients with *PIK3CA* mutations were more likely to benefit from pyrotinib. The clinical effect of concurrent *PIK3CA* mutations may vary according to the HER2 amplification status. The findings must be validated due to the limited number of patients included in present study.

In conclusion, HER2 somatic mutations are common in patients with metastatic breast cancer. The HER2 mutation features differ between patients with HER2 amplification-positive and HER2 amplification-negative breast cancers. A HER2 gene mutation analysis is potentially useful to identify biomarkers of trastuzumab resistance in HER2 amplification-positive patients. HER2 amplification-negative patients carrying HER2 somatic mutations might benefit from pyrotinib. Large-scale prospective studies are needed to verify our findings.

## Methods

### Patients and sample collection

First, we retrospectively analysed the HER2 mutation features in 1184 patients with invasive breast cancer (from two hospitals), who underwent a ctDNA analysis at the Geneplus-Beijing Institute from March 2015 to September 2019. All patients enrolled were female patients with breast cancer who underwent therapy at the National Cancer Center/National Clinical Research Center for Cancer/Cancer Hospital, Chinese Academy of Medical Sciences and Peking Union Medical College and Hunan Cancer Hospital. The clinical characteristics of all patients were collected to determine their correlations with the HER2 mutations. Testing for patients was ordered by the treating physician to identify clinically relevant genomic alterations that might potentially help to determine prognosis or to make treatment decisions. We retrospectively calculated the PFS of HER2 amplification-positive patients administered trastuzumab as the first-line therapy after being diagnosed with metastatic breast cancer to analyse the association between HER2 mutations and the trastuzumab response. Patients were eligible for enrollment if they had a diagnosis of HER2 amplification-positive recurrent or metastatic breast cancer, had not received previous systemic therapy for advanced disease and had received trastuzumab combined with chemotherapy. Patients were excluded if they had received a previous HER2-targeted therapy or endocrine therapy or any previous systemic chemotherapy for advanced disease. Patients who received HER2-targeted therapy combined with endocrine therapy were also excluded.

Then, we conducted a single-arm, prospective, phase-II study of pyrotinib in patients with metastatic HER2 amplification-negative mutation-positive breast cancer (NCT03412383). The study was registered at ClinicalTrialls.gov on 14 January 2018. The test for HER2 mutations was retrospective. Patients who had HER2 mutations in ctDNA and were willing to participate in the present study might be considered to be enrolled. The main inclusion criteria were patients with the ability to understand and willing to sign an Institutional Review Board-approved written informed consent document, at least 18 years of age, with histologically or cytologically confirmed HER2 amplification-negative (0 or 1+ by immunohistochemical staining or non-amplified by fluorescence in situ hybridization), stage IV breast cancer, no standard therapy, at least one measurable disease using the RECIST 1.1 criteria, a Karnofsky performance status > 70, and a life expectancy > 12 weeks. Exclusion criteria included a lack of adequate organ function defined within 2 weeks of registration; pregnant and/or breastfeeding patients; a history of significant cardiac disease, cardiac risk factors or uncontrolled arrhythmias; having a history of uncontrolled paroxysmal diseases, including central nervous system diseases or mental disorders that may alter the understanding and ability to provide informed consent; uncontrolled acute infection; currently receiving any other investigational agents or systemic cancer therapy; allergy to any investigational drug; and any other condition that an investigator considered inappropriate for inclusion in this trial. The primary outcome measure was PFS. AEs, ORR, and CBR were the secondary outcome measures. The patients in whom HER2 somatic mutations were detected were enrolled and received 400 mg of pyrotinib per day.

**Fig. 6:**
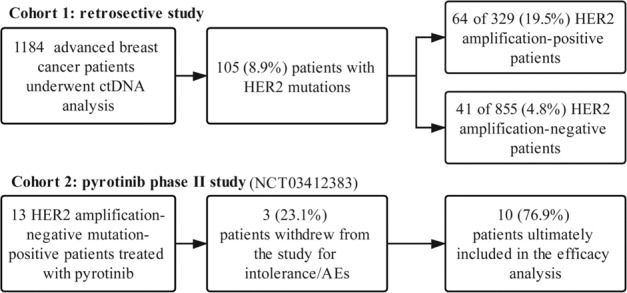
Study flowchart. ctDNA, circulating tumour DNA. AEs adverse events, HER2 human epidermal growth factor receptor 2.

This study, including the retrospective cohort and the prospective cohort, was conducted in accordance with the Declaration of Helsinki and the principles of Good Clinical Practice, and was approved by Regulatory and Ethics Committees of National Cancer Center/National Clinical Research Center for Cancer/Cancer Hospital, Chinese Academy of Medical Sciences and Peking Union Medical College (ref: 16–038/1117 and 17–180/1436). All participants provided written informed consent. The study is compliant with the Guidance of the Ministry of Science and Technology for the Review and Approval of Human Genetic Resources. The study flowchart is shown in Fig. 6.

### Sample collection and DNA extraction

Ten millilitres of peripheral blood were collected from each patient. Peripheral blood samples were collected before receiving trastuzumab therapy or pyrotinib therapy. Peripheral blood samples were collected in Streck tubes (Streck, Omaha, NE, USA) and centrifuged within 72 h, to separate the plasma from the peripheral blood cells. All methods were performed in accordance with the relevant guidelines and regulations, and informed consent was obtained from all patients.

Circulating DNA was isolated using QIAamp Circulating Nucleic Acid Kits (Qiagen, Hilden, Germany). Genomic DNA was isolated from peripheral lymphocytes using the QIAamp DNA Blood Mini Kit (Qiagen). All DNA extractions were performed according to the manufacturer’s protocols and genomic DNA from lymphocytes was sequenced as the normal control sample.

### Target capture and next-generation sequencing

Library preparation, hybrid capture, and circulating DNA and genomic DNA sequencing were performed as previously described^27,28^. A panel of 1021 genes was assayed in the present study (Supplementary Table 4). The targeted region covered ~1.1 Mb of 1021 genes, including all exons of the HER2 gene. DNA sequencing was performed using the HiSeq 3000 Sequencing System (Illumina, San Diego, CA, USA) with 2 × 101 bp paired-end reads.

### Analysis of somatic single-nucleotide variants, indels, and copy number variants

The raw sequencing reads were filtered using NCfilter (version 1.5.0, in-house) to obtain clean data. The clean reads were then aligned to human genome assembly HG19 using Burrows-Wheeler Aligner (version 0.7.15-r1140, also known as BWA)^29^. Duplicate reads of cancer sample derived from PCR amplification were marked using realSeq (version 1.2.0.20170802, in-house application), which was designed to retain reads containing rare events by treating Unique Molecular Indexes, and the normal sample was marked using Picard tools (version 2.6.0-SNAPSHOT, http://broadinstitute.github.io/picard/). Local realignments and base-quality recalibrations were performed using GATK (version 3.6, https://www.broadinstitute.org/gatk/)^30^.

Somatic single-nucleotide variants and small indels were detected using GATK toolkit version 3.6 and realDcaller (version 1.4.1, in-house), and the results of these analyses were merged using NChot (version 0.2.0, in-house) and then annotated to multiple public databases using NCanno (version 0.1.1). Somatic copy number variants were detected using CONTRA (version 2.0.8) software^31^, and the matched peripheral blood cell samples served as matched controls. Significant copy number variations were calculated as the ratio of adjusted depth between cfDNA (hereafter referred to as ctDNA when describing tumour-related gene sequences) and control gDNA. The final candidate variants were all manually verified in the Integrative Genomics Viewer^32^.

### Statistical analysis

PFS was calculated from the date of treatment initiation to the date of disease progression or death from any cause. Patients without an end point (progression or death events) were censored at the date of the last follow-up. Kaplan–Meier survival plots were generated based on gene mutations and curves were compared using log-rank tests. A HER2 subclonal mutation was defined as mutant allele frequency that was <25% of the highest frequency in the sample^33^. All statistical tests performed in the present article were two-sided, and *P*-values < 0.05 were considered significant. All statistical analyses were performed using SPSS version 23.0 (IBM Corporation, Armonk, NY), R v3.6.0 or GraphPad Prism 7.0 (La Jolla, CA).

### Reporting summary

Further information on research design is available in the Nature Research Reporting Summary linked to this article.

## Supplementary information

Supplementary Tables

Supplementary Data 1

Reporting Summary Checklist

## Data Availability

The data generated and analysed during this study are described in the following data record: 10.6084/m9.figshare.12982031^34^. The file “Supplementary Data 1.xlsx” lists somatic mutations identified in 1184 patients and is available together with the figshare data record. The genome sequence data have been deposited in China National GeneBank DataBase (CNGBdb) under the following accession: https://db.cngb.org/search/project/CNP0001305/^35^. The files “Clinical data20200728.xlsx” and “Patient therapy responses and adverse events data.xlsx” contain data on the patients—these datasets are not publicly available in order to protect patient privacy and dataset requests should be sent to the corresponding author.

## Code availability

The code for Figs 2 and 5 were written in R and are available upon request.
