# Supplementary material for: Molecular landscape and efficacy of HER2-targeted therapy in patients with HER2-mutated metastatic breast cancer
Source: NPJ Breast Cancer. 2020 Oct 30;6:59. doi: 10.1038/s41523-020-00201-9 (PMC7603305; doi:10.1038/s41523-020-00201-9)
Supplement: Supplementary file 1 — Supplementary Tables [file 41523_2020_201_MOESM1_ESM.pdf]

Supplementary Table 1 Cox regression analyses of PFS for patients treated with first line trastuzumab

Variable	Univariate analysis		Multivariable analysis	
	**HR (95% CI)**	***P***	**HR (95% CI)**	***P***
Sites of metastasis (as continuous variable)	0.99 (0.80 to 1.24)	0.935		
TP53 status, wild type vs mutant type	1.14 (0.59 to 2.18)	0.700		
PIK3CA status, wild type vs mutant type	0.93 (0.46 to 1.90)	0.847		
PI3K pathway status, wild type vs mutant type	0.87 (0.36 to 2.11)	0.760		
Hormone status, negative versus positive	2.59 (1.30 to 5.16)	0.007	2.64 (1.31 to 5.31)	0.007
HER2 status, wild type vs mutant type	2.59 (1.24 to 5.39)	0.011	2.65 (1.25 to 5.65)	0.011

Abbreviations: PFS: Progression-free survival. HR: hazard ratio. HER2: Human epidermal growth factor receptor 2.

Supplementary Table 2 Characteristics of the 10 patients who received pyrotinib

Patient ID	Age	Histology	Number of metastatic sites	Visceral metastases	Number of chemotherapy line	Number of endocrine therapy line	Previous anti-cancer therapy line	Previous anti-HER2 therapy	Hormone receptor status	Best response achieved	Disease progression	PFS (months)	HER2 mutation
03	51	IDC	1	No	1	3	4	Yes	Positive	SD	1	15.5	p.S310F, p.D769Y
05	55	IDC	3	No	2	1	3	Yes	Positive	PR	1	4.9	p.L755S
06	54	IDC	5	Yes	4	2	6	Yes	Positive	PR	1	5.6	p.Y772_A775dup
07	66	IDC	2	Yes	5	5	10	Yes	Positive	PR	1	4.9	p.D769Y, p.T862A
08	57	IDC	2	No	8	1	9	No	Positive	PD	1	0.7	p.A1076T
09	40	IDC	2	Yes	3	0	3	No	Positive	PD	1	2.7	p.Y772_A775dup
10	37	IDC	1	Yes	3	2	5	No	Positive	SD	1	4.2	p.S310Y, p.L403V
11	47	IDC	3	Yes	2	2	4	No	Positive	CR	0	15.1	p.S310F
12	50	ILC	1	Yes	4	1	5	No	Positive	SD	1	8.2	p.D769Y, p.V777L
13	53	IDC	2	No	4	1	5	No	Positive	PD	1	1.9	p.L767M

Abbreviations: PFS: Progression-free survival. HER2: Human epidermal growth factor receptor 2; CR, complete response; PD, progressive disease; PR, partial response; SD, stable disease.

Supplementary Table 3 Pyrotinib-related adverse events of all grades that occurred in HER2 amplification-negative mutation-positive patients (n=13)

Adverse event	Any Grade		Grade ≥3	
	No.	%	No.	%
Diarrhea	8	61.5	2	15.4
Vomiting	2	15.4	1	7.7
Increased AST	2	15.4	0	0.0
Anorexia	2	15.4	0	0.0
Oral ulceration	1	7.7	0	0.0
Nausea	1	7.7	1	7.7
Increased Creatinine	1	7.7	0	0.0
Increased ALT	1	7.7	0	0.0
Asthenia	1	7.7	0	0.0

Abbreviations: ALT, alanine aminotransferase; ANC, absolute neutrophil count; AST, aspartate aminotransferase; PPE, palmo−plantar erythrodysesthesia; Pyrotinib-related AEs, includes definitely related AEs and probably related AEs.

Supplementary Table 4 List of target region genes

ABCA10	BRSK2	COL5A3	EXOC7	HGF	LRSAM1	NRAS	PRUNE	SLC17A6	TMPRSS2
ABCA8	BRWD3	COL6A5	EXTL3	HID1	LTBP1	NRXN1	PSG2	SLC17A8	TMTC4
ABCB7	BSG	COL6A6	EYA4	HIST1H3B	LUC7L2	NRXN2	PSG5	SLC25A1	TMX3
ABCC8	BTK	COL9A1	EZH2	HLA-DRB1	LUZP4	NT5C3L	PSIP1	SLC25A30	TNFAIP6
ABCF2	BTNL3	COPA	F8	HLA-DRB5	MAEL	NTM	PSMB1	SLC26A3	TNFSF4
ABL1	BTRC	COPG1	F9	HMCN1	MAGI1	NTRK1	PSMB5	SLC2A2	TNN
ABL2	C11orf30	CPA1	FAH	HMHA1	MAN2A1	NTRK3	PSMC4	SLC30A5	TNNT1
ACE	C12orf5	CPSF3	FAM114A2	HNF4A	MAP2	NUDCD2	PSMC6	SLC35B2	TNR
ACER2	C19orf38	CPSF6	FAM131B	HOMER2	MAP2K1	NUP205	PSTPIP1	SLC35B4	TNS3
ACOT11	C1orf112	CRKL	FAM135B	HPS3	MAP2K2	NUP210	PTBP3	SLC38A4	TOP1
ACPP	C1orf35	CRTAM	FAM13C	HPS4	MAP2K4	NUTM1	PTCD3	SLC38A5	TP53
ACSL1	C1QA	CRTAP	FAM157B	HRAS	MAP3K1	NWD1	PTCH1	SLC43A1	TP53BP1
ACSM5	C1S	CRYBG3	FAM177B	HSPA12B	MAP4K1	NXF1	PTCH2	SLC45A1	TPCN1
ACSS3	C20orf112	CSF1R	FAM21A	HSPD1	MAPK1	NXF5	PTEN	SLC4A10	TPH2
ACTL6B	C2orf47	CSMD1	FAM3A	HYDIN	MAPK3	OBP2A	PTGES3L-AARSD1	SLC4A4	TPMT
ADAM23	C2orf62	CSMD3	FAM49A	IBSP	MAPKAPK3	OBP2B	PTGS2	SLC5A1	TPTE
ADAM33	C7orf53	CSN3	FAM49B	IDH1	MAPRE3	OCA2	PTPLAD1	SLC6A5	TRIM33
ADAMTS12	C9orf114	CSNK1E	FAM5C	IDH2	MAST1	ODZ3	PTPN11	SLC8A1	TRIM51
ADAMTS16	C9orf43	CSPP1	FAM86B1	IFT172	MBIP	OR2T4	PTPN13	SLCO1B7	TRIM58
ADAMTS19	CACNA1A	CTCF	FAN1	IGF1R	MBTPS2	OR4A15	PTPRA	SLCO5A1	TRIML1
ADAMTS20	CACNA1D	CTIF	FANCC	IGSF9	MCF2L2	OR4C6	PTPRD	SMARCA4	TRIO
ADAMTS5	CACNA1E	CTNNA2	FASTK	IKBKAP	MCL1	OR5L2	PTPRM	SMARCB1	TRIP11
ADAMTSL1	CADM2	CTNNB1	FAT1	IKBKE	MCOLN2	OR6F1	PYHIN1	SMO	TRMT112

ADD2	CAMKK1	CTSF	FATE1	IL11RA	MDGA2	OSBPL10	QRICH2	SMTN	TRPC5
AGMAT	CAPRIN1	CYP2A13	FBN2	IL13RA2	MDM2	OTOA	RAB1B	SNTG1	TRUB1
AGTPBP1	CARS	CYP3A4	FBXW7	IL1RAPL1	MDM4	OTOGL	RAB3GAP2	SORCS3	TSC1
AHCTF1	CARS2	CYP4A11	FCGR2A	IL27RA	MDN1	OVCH1	RAB6A	SPAG16	TSC2
AK5	CASC4	CYTH4	FCGR2B	IL7R	MED12	P4HB	RAC2	SPATA13	TSGA10
AKR1B10	CASP8	DCLK2	FCGR3A	IMPG1	MED23	PABPC4	RAF1	SPG20	TSKS
AKR1C1	CASP8AP2	DCST1	FDCSP	INHBA	MEFV	PACS2	RALBP1	SPINT1	TSPAN12
AKT1	CASQ2	DDB1	FGFR1	INPP4B	MET	PAEP	RAPGEF2	SPPL2A	TSR2
AKT2	CATSPER2	DDR1	FGFR2	INPP5J	METTL14	PAGE1	RARA	SPPL3	TTF2
AKT3	CBFB	DDR2	FGFR3	IQCA1	METTL5	PALB2	RARB	SPRED1	TTN
ALDH1A3	CBL	DDX24	FGFR4	IRS2	MGAM	PARK2	RASEF	SPTA1	TUBA3C
ALDH2	CBX4	DDX3X	FLCN	ITFG2	MICALL1	PARP4	RB1	SRC	TUBGCP4
ALG5	CCDC155	DEPDC4	FLNC	ITGA8	MID1	PCK2	RBM6	SRRT	TUBGCP5
ALK	CCDC159	DGKK	FLOT2	ITGA9	MIER2	PCLO	RBMX	SSBP3	TYK2
ALX4	CCDC17	DHCR24	FLT1	ITIH1	MITF	PCNT	RCC1	SSH2	TYRP1
AMOT	CCND1	DHDDS	FLT3	ITLN2	MLH1	PCNXL2	REC8	SSPO	U2AF1
ANK2	CCND2	DHX9	FLT3LG	ITM2A	MLH3	PCSK5	REG1B	ST18	U2AF2
ANKRD13D	CCND3	DIAPH1	FLT4	ITPKB	MLL3	PCYT1A	RELN	ST6GALNAC1	UBASH3A
ANKRD20A4	CCNE1	DKC1	FMN2	ITPR1	MLPH	PDCD6	RERE	STAG2	UBE2Q1
ANKRD27	CCT3	DLST	FMNL3	JAK1	MORC1	PDE1C	RET	STAT1	UBE4B
ANKRD28	CCT6B	DMD	FNDC4	JAK2	MORN1	PDE2A	RFWD2	STAT3	UCHL3
ANKRD30A	CD1E	DMXL1	FNIP2	JAK3	MPL	PDE4DIP	RFX3	STAT4	UCK2
ANKRD30B	CD274	DMXL2	FOLH1	KCNAB2	MRPL1	PDGFRA	RHEB	STAT6	UGT8
ANKRD36B	CD300LF	DNAH10	FOXA1	KCNH6	MRPL24	PDGFRB	RHOA	STK11	ULK3
ANO2	CD5L	DNAH5	FOXJ2	KCNQ2	MRPS18B	PDIA5	RICTOR	STK11IP	UMOD

AP1B1	CD9	DNAH9	FOXL2	KDM4A	MS4A1	PDILT	RNF215	STK31	UNC13A
AP1G2	CD97	DNAJC11	FRG1	KDM6A	MSH2	PDK1	RNF219	STX3	UNC13D
AP3B1	CD99	DNAJC9	FRG2B	KDR	MSH3	PDRG1	RNF43	SULT1A4	UNC5D
APAF1	CDH1	DNMT3A	FRMD4A	KEAP1	MSH6	PEX6	ROCK1	SUPT5H	USP12
APC	CDH18	DNTTIP1	FRMPD2	KIAA0195	MSI1	PGAP1	ROS1	SUPT6H	USP34
APLP2	CDH24	DOCK11	FRMPD4	KIAA0226	MTA2	PHACTR3	RPL22	SYCP2L	USP39
APMAP	CDH26	DOCK3	FSD2	KIAA0319	MTM1	PHF20L1	RPL36A	SYK	USP45
APPL2	CDK11A	DOT1L	FSHR	KIAA0922	MTOR	PHYH	RPS5	SYNE1	USP48
AQP12A	CDK12	DPP10	FUBP1	KIAA1191	MTR	PI4KB	RPS6KA1	SYNE2	VAV1
AR	CDK13	DPP4	FUNDC1	KIAA1199	MTTP	PIK3CA	RPS6KB1	SYNJ2	VEGFA
ARAF	CDK14	DRGX	GAB2	KIAA1211L	MUC5B	PIK3CB	RPTOR	TAF1B	VEZF1
ARFGAP1	CDK18	DUOX1	GAB3	KIF13A	MUS81	PIK3R1	RPUSD4	TAF6	VHL
ARFRP1	CDK19	DYSF	GABRD	KIF1B	MYB	PIK3R2	RREB1	TARBP1	VILL
ARHGAP35	CDK4	DZANK1	GAD2	KIF26B	MYBPC2	PIP4K2C	RRP7A	TBC1D1	VIT
ARHGAP40	CDK6	ECHDC1	GALNT13	KIF5B	MYC	PIP5K1C	RUNDC3A	TBC1D21	VPS13A
ARHGEF1	CDK8	EDN1	GALNT14	KIFAP3	MYCBP2	PIWIL1	RUNX1	TBC1D3	VPS33B
ARHGEF7	CDKN1A	EEF1A1	GATA3	KIFC1	MYD88	PKD1L2	RYR2	TBC1D5	VSIG4
ARNTL	CDKN1B	EFCAB5	GFRAL	KIR2DL3	MYH15	PKHD1	RYR3	TBL1X	WAS
ARPC4-TTLL3	CDKN2A	EFCAB6	GIGYF1	KIR3DL3	MYH2	PKLR	SAFB2	TBP	WASL
ASH2L	CDKN2B	EFCAB7	GINS4	KIT	MYH4	PLAC8	SAG	TBX15	WDR44
ASTN1	CDS1	EFHA2	GIPR	KLHL1	MYH8	PLCB4	SAGE1	TBX22	WDR52
ASXL2	CEACAM20	EFNA5	GKN2	KLHL14	MYH9	PLCZ1	SAMD8	TBX3	WDR62
ATAD2B	CECR2	EGFR	GLB1L3	KLK1	MYL5	PLEC	SCN10A	TCF20	WDR66
ATG9B	CELA2B	EIF1AX	GLYR1	KMT2B	MYL6	PLK2	SCN3A	TCF4	WDR72
ATM	CGN	EIF2B5	GMDS	KMT2C	MYLK2	PLOD3	SCN7A	TCP10	WDTC1

ATP10B	CHD3	EIF2C2	GNA11	KRAS	MYO3A	PLXNA1	SCN9A	TCP11	WLS
ATP10D	CHD4	EIF3E	GNAQ	KRT2	MYOM1	PMS1	SDK2	TEK	WSCD2
ATP12A	CHD6	EIF3I	GNAS	KRT9	NACAD	PMS2	SEC14L4	TERT	WWP2
ATP2C1	CHEK1	EIF4ENIF1	GNPTAB	KRTAP5-5	NARF	POLDIP2	SEC24B	TESC	XBP1
ATP6V0A2	CHEK2	EIF4H	GOLGA4	KTN1	NAT10	POLE	SEH1L	TEX35	XPO1
ATP8B2	CHI3L1	ELAVL3	GPAT2	L3MBTL1	NAV3	POLR2J	SELP	TFDP1	XPO4
ATR	CISD3	ELL3	GPATCH2	LARP1	NBPF1	POLR3B	SEMA6A	TGDS	XPO5
ATXN2	CLCN7	EMID2	GPR114	LCN10	NBPF10	POLR3GL	SEPT12.	TGM2	XRCC1
ATXN7L2	CLEC16A	ENPP2	GPR125	LCT	NCF2	POLRMT	SERPINA7	TGM5	ZAP70
AURKA	CLINT1	ENTPD6	GPR133	LCTL	NCKAP1	POM121L12	SETD1B	THBS2	ZBTB8OS
AURKB	CNGB3	EPB41L2	GPR144	LETM1	NCOR1	POTEG	SETD2	THEM5	ZC3H13
AXL	CNKSR2	EPB41L4B	GPS2	LGALS13	NCOR2	PPA1	SF1	THOC1	ZC3H7B
BAP1	CNOT3	EPHA2	GRIA3	LILRB3	NEK5	PPDPF	SF3B1	THSD7A	ZDHHC11
BAX	CNOT4	EPHA3	GRIK2	LILRB4	NELL1	PPEF1	SF3B14	THSD7B	ZFC3H1
BBS9	CNTN1	EPHA5	GUCY1A3	LIPN	NF1	PPFIBP2	SF3B3	TIMD4	ZFR
BCAS1	CNTN4	EPHB1	GUCY2C	LMAN1L	NF2	PPIL2	SGCZ	TIMM44	ZMYM4
BCAS2	CNTN5	EPS8L3	GYLTL1B	LMBR1L	NFE2L2	PPP1R17	SGIP1	TIMP3	ZNF143
BCL2	CNTNAP3B	ERBB2	HAAO	LPCAT4	NIPBL	PPP4R4	SGK1	TJP3	ZNF350
BCL2L11	CNTNAP5	ERBB3	HAP1	LPHN3	NLGN3	PQBP1	SGPL1	TLE1	ZNF385A
BCR	COASY	ERBB4	HAUS5	LRBA	NLRC3	PREB	SH2D3A	TLL1	ZNF414
BLOC1S1	COL14A1	ERCC1	HAUS6	LRP1B	NLRP4	PREX2	SH3BGR	TMC2	ZNF512B
BMPR1B	COL16A1	ERG	HCN1	LRP2	NMI	PRKAA1	SH3PXD2A	TMED8	ZNF541
BRAF	COL19A1	ESD	HDAC1	LRP4	NOP2	PRKACA	SHISA4	TMEM104	ZNF563
BRCA1	COL1A1	ESR1	HDAC4	LRRC16B	NOS1	PRKAG3	SI	TMEM120B	ZNF614
BRCA2	COL25A1	ETNK2	HDAC6	LRRC2	NOS2	PRKCD	SIDT2	TMEM132D	ZNF687

BRD2	COL4A5	ETV6	HEATR7B2	LRRC7	NOTCH1	PRKDC	SIK3	TMEM145	ZNF705B
BRD3	COL4A6	EXOC4	HECTD4	LRRC72	NOTCH2	PRKX	SIM1	TMEM247	ZNF705G
BRD4	COL5A1	EXOC5	HECW1	LRRD1	NOTCH3	PRRX1	SIM2	TMEM80	ZNF711
BRF1	COL5A2	EXOC6	HECW2	LRRFIP2	NOTCH4	PRSS1	SLC13A3	TMEM87A	ZNF804B
									ZSWIM8

Supplementary Data 1. Somatic mutations identified in 1184 patients. (separate .xlsx file)
