# Supplementary material for: Molecular landscape and efficacy of HER2-targeted therapy in patients with HER2-mutated metastatic breast cancer
Source: NPJ Breast Cancer. 2020 Oct 30;6:59. doi: 10.1038/s41523-020-00201-9 (PMC7603305; doi:10.1038/s41523-020-00201-9)
Supplement: Supplementary file 3 — Reporting Summary Checklist [file 41523_2020_201_MOESM3_ESM.pdf]

nature research

Corresponding author(s): Fei Ma

Last updated by author(s): Oct 3, 2020

Reporting Summary

Nature Research wishes to improve the reproducibility of the work that we publish. This form provides structure for consistency and transparency in reporting. For further information on Nature Research policies, see our Editorial Policies and the Editorial Policy Checklist.

Statistics

For all statistical analyses, confirm that the following items are present in the figure legend, table legend, main text, or Methods section.

n/a	Confirmed	
☐	☒	The exact sample size (n) for each experimental group/condition, given as a discrete number and unit of measurement
☒	☐	A statement on whether measurements were taken from distinct samples or whether the same sample was measured repeatedly
☐	☒	The statistical test(s) used AND whether they are one- or two-sided<br>*Only common tests should be described solely by name; describe more complex techniques in the Methods section.*
☐	☒	A description of all covariates tested
☐	☒	A description of any assumptions or corrections, such as tests of normality and adjustment for multiple comparisons
☐	☒	A full description of the statistical parameters including central tendency (e.g. means) or other basic estimates (e.g. regression coefficient) AND variation (e.g. standard deviation) or associated estimates of uncertainty (e.g. confidence intervals)
☐	☒	For null hypothesis testing, the test statistic (e.g. F, t, r) with confidence intervals, effect sizes, degrees of freedom and P value noted<br>*Give P values as exact values whenever suitable.*
☒	☐	For Bayesian analysis, information on the choice of priors and Markov chain Monte Carlo settings
☒	☐	For hierarchical and complex designs, identification of the appropriate level for tests and full reporting of outcomes
☒	☐	Estimates of effect sizes (e.g. Cohen's d, Pearson's r), indicating how they were calculated

Our web collection on statistics for biologists contains articles on many of the points above.

Software and code

Policy information about availability of computer code

Data collection	No software was used.
Data analysis	All statistical analyses were performed using SPSS version 23.0 (IBM Corporation, Armonk, NY), R v3.6.0 or GraphPad Prism 7.0 (La Jolla, CA).

For manuscripts utilizing custom algorithms or software that are central to the research but not yet described in published literature, software must be made available to editors and reviewers. We strongly encourage code deposition in a community repository (e.g. GitHub). See the Nature Research guidelines for submitting code & software for further information.

Data

Policy information about availability of data

All manuscripts must include a data availability statement. This statement should provide the following information, where applicable:

- Accession codes, unique identifiers, or web links for publicly available datasets
- A list of figures that have associated raw data
- A description of any restrictions on data availability

The datasets generated during and/or analysed during the current study are available from the corresponding author on reasonable request.

Field-specific reporting

Please select the one below that is the best fit for your research. If you are not sure, read the appropriate sections before making your selection.

☒ Life sciences ☐ Behavioural & social sciences ☐ Ecological, evolutionary & environmental sciences

For a reference copy of the document with all sections, see nature.com/documents/nr-reporting-summary-flat.pdf

Life sciences study design

All studies must disclose on these points even when the disclosure is negative.

Sample size	We retrospectively analysed the HER2 mutation features of 1184 patients with invasive breast cancer.
Data exclusions	No data was excluded in the present study.
Replication	This is a clinical study, no experimental data was included.
Randomization	This study included a retrospective study and a phase II single arm clinical trail. The randomization was not relevant to our study.
Blinding	This study included a retrospective study and a phase II single arm clinical trail. The randomization was not relevant to our study.

Reporting for specific materials, systems and methods

We require information from authors about some types of materials, experimental systems and methods used in many studies. Here, indicate whether each material, system or method listed is relevant to your study. If you are not sure if a list item applies to your research, read the appropriate section before selecting a response.

Materials & experimental systems

n/a	Involved in the study	
☒	☐	Antibodies
☒	☐	Eukaryotic cell lines
☒	☐	Palaeontology and archaeology
☒	☐	Animals and other organisms
☐	☒	Human research participants
☐	☒	Clinical data
☒	☐	Dual use research of concern

Methods

n/a	Involved in the study	
☒	☐	ChIP-seq
☒	☐	Flow cytometry
☒	☐	MRI-based neuroimaging

Human research participants

Policy information about studies involving human research participants

Population characteristics	In total, 1184 female patients with metastatic breast cancer were enrolled. The median age was 46 years old.
Recruitment	We retrospectively analysed the HER2 mutation features in 1184 patients with invasive breast cancer (from 2 hospitals) who underwent a ctDNA analysis at the Geneplus-Beijing Institute from March 2015 to September 2019. All patients enrolled were female patients with breast cancer who underwent therapy at the National Cancer Center/National Clinical Research Center for Cancer/Cancer Hospital, Chinese Academy of Medical Sciences and Peking Union Medical College and Hunan Cancer Hospital.
Ethics oversight	This study was conducted in accordance with the Declaration of Helsinki and the principles of Good Clinical Practice and was approved by Regulatory and Ethics Committees of National Cancer Center/National Clinical Research Center for Cancer/ Cancer Hospital, Chinese Academy of Medical Sciences and Peking Union Medical College (ref: 16-038/1117 and 17-180/1436). All participants provided written informed consent.

Note that full information on the approval of the study protocol must also be provided in the manuscript.

Clinical data

Policy information about clinical studies

All manuscripts should comply with the ICMJE guidelines for publication of clinical research and a completed CONSORT checklist must be included with all submissions.

Clinical trial registration	NCT03412383

Study protocol	The study protocol could be found in ClinicalTrails.gov.
Data collection	This is an investigator-initiated-clinical trail, and data was collected by investigator of the study.
Outcomes	The primary outcome measure was PFS. AEs, ORR, and CBR were the secondary outcome measures.
